# Preparation and Physico-Chemical Characterization of OSA-Modified Starches from Different Botanical Origins and Study on the Properties of Pickering Emulsions Stabilized by These Starches

**DOI:** 10.3390/polym15030706

**Published:** 2023-01-31

**Authors:** Fengchao Zhou, Mingyang Dong, Jianhui Huang, Guorong Lin, Jie Liang, Shibin Deng, Chenqi Gu, Qingyu Yang

**Affiliations:** 1 Fujian Provincial Key Laboratory of Ecology-Toxicological Effects & Control for Emerging Contaminants, College of Environmental and Biological Technology, Putian University, Putian 351100, China; 2 College of Grain Science and Technology, Shenyang Normal University, Shenyang 110034, China

**Keywords:** rice starch, tapioca starch, oat starch, Pickering emulsions, OSA, stability

## Abstract

Native starch (NS) from different botanical origins (native rice/tapioca/oat starch, NRS/NTS/NOS) were hydrophobically modified by octenyl succinic anhydride (OSA), and the octenyl succinic (OS) groups were successfully introduced in the starch molecules which obtained OS-starch (OSRS, OSTS and OSOS) with different levels of modification (0.5%, 1.0%, 1.5%, 2.0%, 2.5%, 3.0%) and degree of substitution (DS). The structural properties of the OS-starch, such as granule size, crystal, wettability and morphology were studied, and the OS-starch was used as particulate stabilizers to produce oil-in-water (O/W) Pickering emulsions. The emulsion index, droplet size distribution and microstructures of Pickering emulsions produced by different OS-starches were compared. OSA modification had almost no effect on the morphology or crystal structure types of three kinds of NS and OS-starch but markedly increased the contact angle and particle size distribution of OSRS, OSTS and OSOS. Esterification reaction of OSA and starch mainly occurred in amorphous regions of starch, and the OSA significantly improved the emulsifying capacity of OSRS, OSTS and OSOS granules and thus stabilized emulsions formed at higher levels (2.5% and 3.0%) of modification of OS-Starch exhibited better stability; the ability of OS-starch to stabilize Pickering emulsion was 3.0% OSRS > 3.0% OSOS > 3.0% OSTS, respectively. Observation and structural properties analysis of OS-starch granules and Pickering emulsion droplets showed that the number and thickness of the starch granules on the oil-water interface of the emulsion droplets increased with improvement of the OSA modification level, and an aggregation state was formed between the OS-starch granules, which was also enhanced with the OSA modification levels. These were all necessary for the Pickering emulsion stabilized by starch granules to remain in a steady state.

## 1. Introduction

Recently, there has been more research on Pickering emulsions stabilized by particulate emulsifier from food raw materials [1,2]. The Pickering emulsion stabilized by solid small particles can stabilize the emulsion droplets by dual wettability of the particles in both oil and water phases. Compared with the traditional emulsifiers, the edible particulate emulsifiers have a stronger resistance to the aggregation, which can form a spatial barrier between the emulsion droplets to prevent droplet aggregation [3,4]. Current studies have demonstrated that starch granules could be used as particulate stabilizers in Pickering emulsion [5,6,7]. Generally, the emulsifying capacities of most native starch (NS) is relatively weak and not hydrophobic, which is not easy to adsorb to the oil-water interface in the emulsification process, but it can be modified by octenyl succinic anhydride (OSA) to improve its hydrophobicity in order to improve its emulsification capacity [8].

OSA belongs to a class of chain-enyl succinic, where the chain-enyl group in the chain-enyl succinic structure can be an alkyl, alkenyl, aromatic alkyl or aryl group, or a lipophilic chain containing 5–18 carbon atoms. Octenyl succinic starch (OS-starch) is a recognized safe emulsifier among biological macromolecular emulsifiers, which has characteristics of being a colorless, odorless, inexpensive, non-allergic and approved food additive and excipient with a degree of modification lower than 3% based on the dry weight of starch, with no limit on application [9,10]. OS-starch, produced by industrialization and called pure glue, is usually obtained from the esterification reaction by native starch with OSA in a mild alkaline aqueous solution, which has the better emulsification stability in the oil-in-water (O/W) emulsion system and has been widely used in food, the chemical industry and other fields [11,12,13,14]. The hydroxyl groups at the positions C-2, C-3, and C-6 of the starch molecule glucose units can be replaced by the OS groups under alkaline conditions for an esterification reaction [9]. The hydrophobic OS groups bind to the molecular structure of the starch to convert the native starch into both hydrophilic and hydrophobic amphiphilic particles.

At present, OS-starch is widely used in the food industry, however the use of oat, rice and tapioca starches is unconventional, and the Pickering emulsion prepared by OS-starch has demonstrated its unique advantages and an important role in many fields [15,16], but it was rarely used in the field of meat products. In this study, native rice, tapioca and oat starch (NRS, NTS and NOS) were modified with OSA to obtain OS-starch (OSRS, OSTS and OSOS) with different OSA modification levels and degrees of substitution (DS). The OSRS, OSTS and OSOS samples were characterized and used as particulate emulsifiers to stabilize Pickering emulsions, and the properties of the Pickering emulsions were investigated. The results obtained from our work would be useful in the design and development of OS-starch-based Pickering emulsifiers and would provide a theoretical basis for the application of Pickering emulsion based on OS-starch in food and especially in emulsified comminuted meat products.

## 2. Materials and Methods

### 2.1. Materials

The following are the materials and their vendors: NRS (Shanghai Yuanye Biotechnology Co., Ltd., Shanghai, China); NTS (Nanjing Ganzhiyuan Sugar Industry Co., Ltd., Nanjing, China); raw oats (Putian Yonghui Supermarket, Putian, China); OSA (Shanghai Aladdin Biochemical Technology Co., Ltd., Shanghai, China); and sunflower seed oil (Jiage Investment (China) Co., Ltd., Shanghai, China). All other chemicals were of analytical grade.

### 2.2. Extraction of NOS

Extraction of NOS was according to the procedure of Falsafi et al. [17], with some modification. Raw oats were crushed and oat powder was obtained. The oat powder and distilled water slurry were prepared in a ratio of 1:6 (*m/V*) and adjusted to pH10 with an NaOH solution (1.0 mol/L), a 25 °C constant temperature water bath and magnetic stirring for 90 min. After stirring, the fiber was removed by using a 100-mesh sieve filter, and the filtrate was centrifuged at 3000 r/min for 15 min; precipitate was washed to pH7 using HCl solution (0.0025 mol/L) and centrifuged again at 3000 r/min for 15 min; precipitate was washed with distilled water and finally centrifuged at 3000 r/min for 15 min. The precipitate was dried at 35 °C for 36 h, crushed and passed through a 100-mesh sieve to obtain NOS.

### 2.3. OSA Modification of Starch Granules

Native starches (NRS, NTS, NOS) were esterified using OSA according to the procedure of Marefati et al. [9], with some modification. A total of 50.0 g of the starch was suspended in 200.0 g of distilled water; the pH was adjusted to 8.2–8.4 by titration with a 0.5 M NaOH solution and maintained constant during the reaction. Then, a solution of OSA in absolute ethyl alcohol (100 mg OSA/mL solution) was added within 120 min and the temperature was kept constant (35.0 ± 0.5 °C). The total amount of added OSA was varied (0.5%, 1.0%, 1.5%, 2.0%, 2.5% and 3.0%) in relation to the dry matter of the starches. The reaction finished after 180 min, then the pH was adjusted to 6.5 by 0.5 M HCl solution. The acquired suspension was centrifuged at 3500 rpm for 15 min and was washed twice with distilled water and twice with 95% ethanol; then, the obtained OS-starch was dried in an oven at 40 °C for 24 h, ground and passed through a 100-mesh sieve (150 μm). Finally, three kinds of OS-starch samples (OSRS, OSTS, OSOS) with modification levels of 0.5%, 1.0%, 1.5%, 2.0%, 2.5%, and 3.0% were obtained for further analysis.

### 2.4. Degree of Substitution (DS) and Reaction Efficiency (RE)

The DS of OS-starch was determined by titration method according to Ruan et al. [18], with minor modification. The OS-starch (2.5 g, dry weight) was suspended in 25 mL of 2.5 M HCl-ethanol solution with constant stirring for 30 min. Then, 50.00 mL of 95% ethanol was added to the reaction system, stirring continuously for 10 min and filtered to obtain precipitate. The precipitate was moved into the Bouchell funnel and washed with 90% ethanol to remove the chloride ions (tested with 0.1 M AgNO_3_), then the precipitate was suspended in 300 mL of deionized water, heated in a boiling water bath with constant stirring for 20 min, and then titrated with 0.1 M NaOH. Phenolphthalein was used as an indicator, and the color of the solution changed from transparent to pink to the end point. The DS was calculated by the following formula:

(1) $$DS=\frac{162\times \left(A\times M\right)/W}{1000-209\times \left(A\times M\right)/W}$$

where *A* is the volume (mL) of NaOH required for sample titration, *W* is the starch weight (g), *M* is the molarity of NaOH, 162 is the molecular weight of the glucose unit, and 209 is the molecular weight of octenyl succinic group minus the molecular weight of hydrogen.

The RE was calculated as follows:

(2) $$RE=\frac{209\times DS\times m_{starch}}{162\times m_{OSA}}\times 100\%$$

where *m_starch_* and *m_OSA_* are the quality (g) of the starch and *OSA* used in the reaction, respectively.

### 2.5. Particle Size Distributions of Starch Granule-Water Dispersion Solution

The particle size distribution (PSD) of the starch sample was measured using a laser diffraction particle size analyzer (Malvern Mastersizer 2000; Malvern Instruments Ltd., Worcestershire, UK). The refractive index of the starch phase was 1.54 and the water phase was 1.33 [9]. The result was recorded as the median mean particle size d(0.5) and volume-average droplet size d[4,3].

### 2.6. Contact Angle

The contact angle of starch samples was determined using an OCA 20 (Dataphysics instruments GmbH, Filderstadt, Germany), following the method described by Dai et al. [19]. The starch powders were pressed to obtain particle-based tablets (12 mm in diameter and 2 mm in thickness). A drop of Milli-Q water (2 μL) was then lightly dripped onto the surface of the tablets using a high-precision injector. After equilibrium was reached, the droplet was photographed and acquire the contact angle by the software. Measurements were performed at least three times.

### 2.7. Microstructural Observation of the Starch Granules

#### 2.7.1. Polarized Light Microscopy (PLM)

The crystal structures of starch samples were observed using a polarized microscope (BS203IP, Chongqing Photoelectric Instrument Co., Ltd., Chongqing, China). The starch samples were regulated in a certain proportion to form an emulsion (10%, *w*/*v*). A drop of the liquid was placed on a glass slide, covered with a coverslip, and placed on the objective table. Then, the sample was viewed using objective and eyepiece magnifications of 40× and 10× or 20× and 10×, respectively.

#### 2.7.2. Scanning Electron Microscopy (SEM)

The morphologies of the starch granules were observed by SEM (Regulus8100, Hitachi, Tokyo, Japan). The starch samples were suspended in acetone at 1% (*w*/*v*). Each sample was spread directly onto the surface of the stub and dried in an oven at 32 °C for 1 h. Then, all of the samples were coated with gold and examined in the SEM under an acceleration voltage of 5 kV.

### 2.8. Fourier Transform Infrared Spectroscopy (FT-IR)

The changes in chemical structure of starch samples were qualitatively analyzed using FT-IR (NICOLET 5700, Thermo Electric Corporation, Waltham, MA, USA). Samples were prepared by grinding the finely powdered starch with KBr (1:100, *w*/*w*) and scanned in the wave number range of 400–4000 cm^−1^.

### 2.9. X-ray Diffraction (XRD)

X-ray diffractograms of the starch samples were determined using an X-ray diffractometer (D8 ADVANCE, Bruker, Billerica, MA, USA). The scanning region of the diffraction ranged from 5°to 50°with a target voltage of 40 kV, current of 40 mA and scan speed of 2°/min. The relative crystallinity (RC) of the starch granules was calculated according to the following equation:

(3) $$RC=\frac{A_{c}}{A_{c}+A_{a}}\times 100\%$$

where *A_c_* is the crystalline area, and *A_a_* is the amorphous area on the X-ray diffractograms.

### 2.10. Preparation of Starch-Based Pickering Emulsions

The starch-based Pickering emulsions were prepared as described by Marefati et al. [9], with some modifications. Using sunflower seed oil stained with Sudan red as dispersed phase; phosphate buffer (95%, 50 mM, pH6.2, 0.6 M NaCl) as continuous phase; and starch granules from native and different modification levels (200 mg of starch/mL oil) of 0.5–3.0% OSA to stabilize the emulsions, 5% *v*/*v* oil-in-water starch granules stabilized emulsions were prepared. The emulsions were homogenized using a rotor-stator high shear homogenizer (FA25-D, Fluke, Everett, WA, USA) at 22,000 rpm for 60 s, and the starch-based Pickering emulsions were obtained.

### 2.11. Emulsion Index (EI) Test

**Figure 1:**
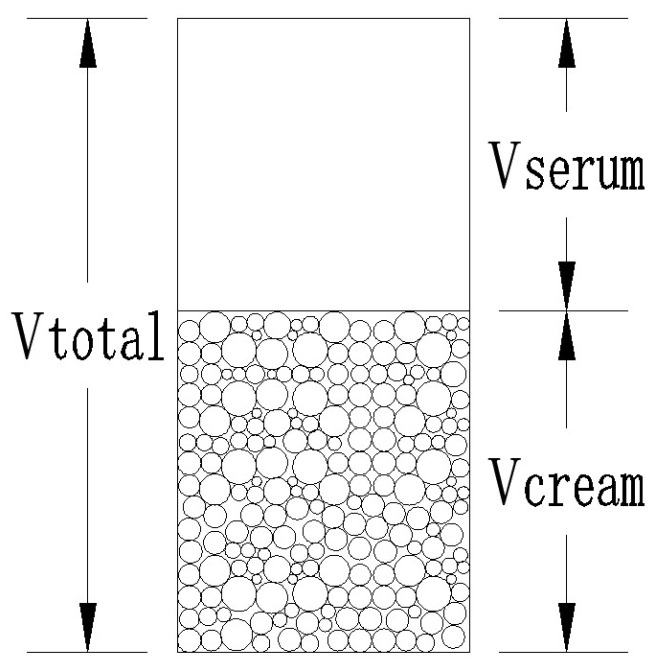
Schematic representation of EI. Vserum: volume of clear liquid after homogenization; Vcream: volume of the emulsified layer after homogenization; Vtotal: volume of total emulsion.

The emulsion index (EI) refers to the proportion of the emulsified layer of the emulsion relative to the total volume of the emulsion. The Pickering emulsions obtained from 2.10 were transferred to 25 mL scale tubes, 25 °C standed for 1 d, 7 d and 15 d, and the observed emulsified layer volume was recorded (Figure 1). The EI values were calculated according to the following the equation:

(4) $$EI=\frac{V_{\mathrm{cream}}}{V_{\mathrm{total}}}\times 100\%$$

### 2.12. Droplet Size Distribution of Pickering Emulsions

The droplet size distribution (DSD) of Pickering emulsions was measured using a laser diffraction particle size analyzer (Malvern Mastersizer 2000; Malvern Instruments Ltd., Worcestershire, UK). The refractive index of the starch phase was 1.54, the water phase was 1.33 and the oil phase was 1.52. The result was recorded as the median mean particle size d(0.5) and volume-average droplet size d[4,3].

### 2.13. Microscopic Observation of Pickering Emulsions

The microstructure of the starch-based Pickering emulsions was observed using a polarized microscope (BS203IP, Chongqing Photoelectric Instrument Co., Ltd., Chongqing, China) and photographed to evaluate the droplet size and distribution state. Each sample was placed on a clean dry microscope slide and covered with a cover slip prior to analysis.

### 2.14. Statistical Analysis

For each batch of samples, all specific experiments were carried out in triplicate (triplicate observations). All data were analyzed using the General Linear Models procedure in the Statistix 8 version 8.1 software package (Analytical Software, St. Paul, MN, USA). Analysis of variance was performed to determine the significance of the main results. Significant differences (*p* < 0.05) were identified using Tukey procedures.

## 3. Results

### 3.1. Preparation and Physico-Chemical Properties of OS-Starch

#### 3.1.1. Degree of Substitution (DS) and Reaction Efficiency (RE)

**Figure 2:**
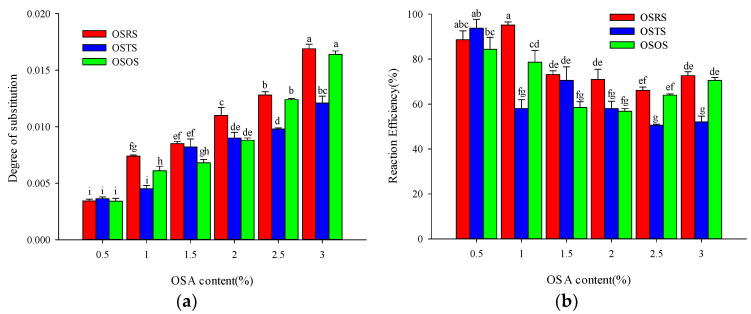
DS (**a**) and RE (**b**) values of OS-starch. The data means average value (*n* = 3); the different letters (a–i) means differ significantly (*p* < 0.05), the same indicates that the difference is not significant (*p* > 0.05).

As shown in the Figure 2a, the DS values of OSRS (0.0034–0.0169), OSTS (0.0038–0.0121) and OSOS (0.0034–0.0164) all significantly increased with the increased level (0.5–3.0%) of OSA modification, and Figure 2b shows the RE values of the corresponding OS-starch. The increased OSA concentration also improved the probability of contact between starch molecules and OSA, which promoted the progress of esterification substitution reaction and more hydroxyl groups on starch molecules were replaced, thus the DS values were increased. At present, the quality standard of food additives stipulates that the degree of OS-starch modification is less than 3% (the amount of modifier used is less than 3% of the quality of starch dry base), and the DS when used as a food additive does not exceed 0.02 [10,20]. The modification levels and DS values of OS-starch obtained in this study were all controlled within the required limits of food additives; it can be directly applied as a particle emulsifier in the food system. The difference between the DS and RE values of different plant-derived OS-starch were related to starch particle size as well as particle structure, and it will be discussed in the following results.

#### 3.1.2. Morphologies and Particle Size Distribution (PSD)

**Figure 3:**
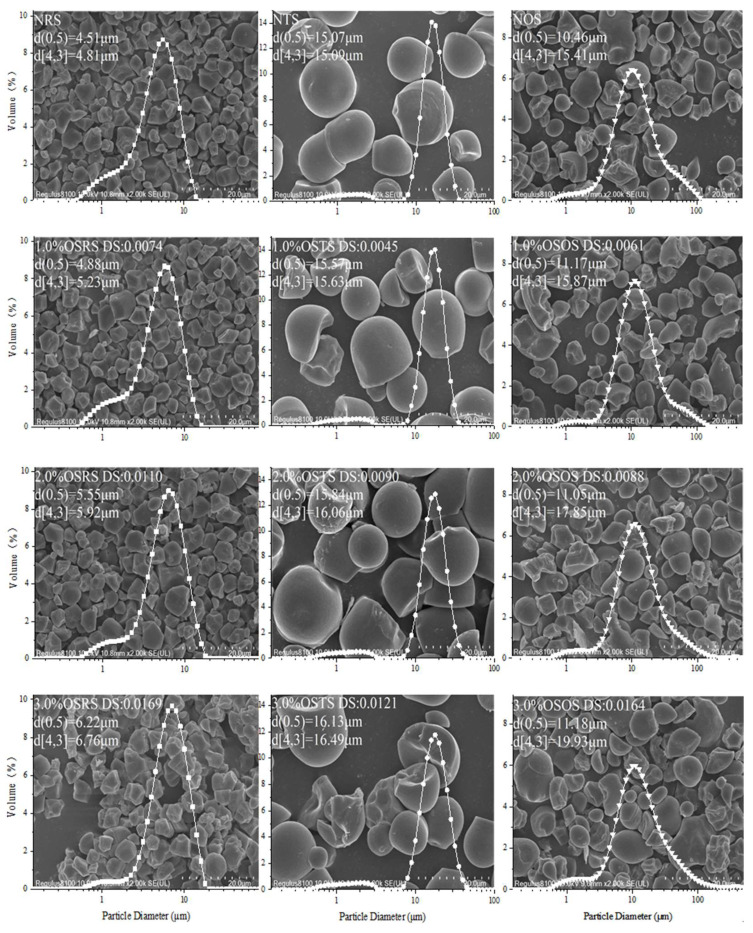
SEM and PSD of NS and OS-starch.

Figure 3 shows the scanning electron micrographs and PSD of NS (NRS, NTS, NOS) and OS-starch (OSRS, OSTS, OSOS). NRS were irregular polygon particles with a relatively uniform size, d(0.5) value of 4.51 μm and a unimodal distribution (particle size range of 0.50–14.15 μm). NTS were mainly spherical large particles and a small number of unspherical small particles, with a d(0.5) value of 15.07 μm and a bimodal distribution (large particle size range 6.32–35.56 μm, small particle size range 0.44–3.16 μm). The particle size range of NOS was relatively wide (0.63–112.46 μm), and mainly oval small particles or a small number of irregular shapes of large particles, with a d(0.5) value of 10.46 μm and showing a single peak distribution. These were in line with the results reported in the literature [9,14].

The d(0.5) and d[4,3] values of three types of OS-starch were significantly improved compared with that of NS after being modified by OSA and increased with the DS value. The increase in d(0.5) and d[4,3] values indicates that a certain degree of aggregation of the OS-starch granules occurred in the aqueous solution, and a greater DS value reflected a stronger aggregation of the OS-starch granules [16]. Aggregation of the OS-starch granules were clearly observed in the SEM photos of the 3.0% OSRS and 3.0% OSTS. Jiang et al. [21] showed that when the surface hydrophobic activity of OSA-modified taro starch granules was significantly improved (under high DS conditions), a certain degree of aggregation occurred in aqueous solution due to the significantly enhanced hydrophobic interaction. When the OS-starch granules were dispersed in an aqueous solution, the hydrophobic OS groups on the particle surface enhanced the hydrophobic interaction between the particles with an increased DS value, thus a certain degree of aggregation of the OS-starch granules occurred. Rayner et al. [22] suggested that the aggregated structure resulting from the aggregation of OSA quinoa starch granules (4.66%OSA) helps to create a thick multi-granule layer at the surface of drops, thereby increasing the stability of formed emulsions by the OSA quinoa starch granules. Therefore, we hypothesized that the adsorption behavior of OSRS, OSTS and OSOS as granular emulsifiers at the emulsion oil-water interface would also be affected by the DS values.

#### 3.1.3. Structural Properties

**Figure 4:**
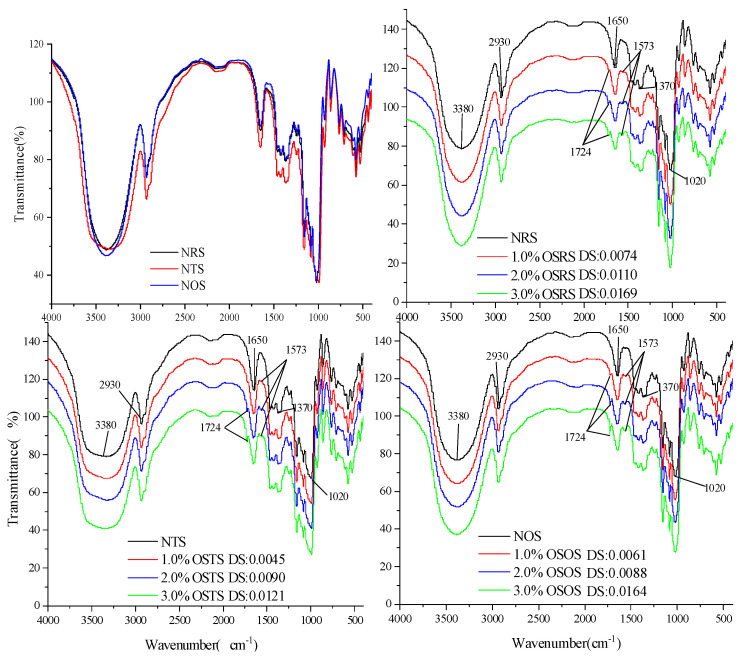
FT−IR spectra of NS and OS-starch.

The structural unit of the starch molecules is α -D-pyran glucose, and the main characteristic absorption peak of the infrared spectrum comes from the hydroxyl groups on C_2_, C_3_, and C_6_, as well as the circular structure of the glucose molecules. It is observed from Figure 4 that the NRS, NTS and NOS have the same characteristic absorption peaks at 3380 cm^−1^, 2930 cm^−1^, 1650 cm^−1^, 1370 cm^−1^ and 1020 cm^−1^. Among them, the larger peaks occurring at 3380 cm^−1^ were generated by O-H expansion vibration; the peaks occurring at 2930 cm^−1^ were generated by C-H expansion vibration; the peaks occurring at 1650 cm^−1^, 1370 cm^−1^ and 1020 cm^−1^ were generated by C-O [23]. New characteristic absorption peaks of the infrared spectra of OSRS, OSTS and OSOS appear at 1724 cm^−1^ and 1573 cm^−1^ after being modified by OSA, which correspond to the C = O expansion vibration of the ester group and the asymmetric expansion vibration of the RCOO-group [24], respectively, and the intensity of the new characteristic peaks enhanced with the increasing of DS value. These indicated that the OS groups replaced part of the hydroxyl groups on the NS molecule and connected to the hydroxyl group on the glucose unit as ester bonds, which was a marker of the successful esterification of OSA with NRS, NTS and NOS. Song [14] and Wang [11] obtained similar results by performing a hydrophobic modification with wheat starch, rice starch, and corn starch using OSA, respectively.

**Figure 5:**
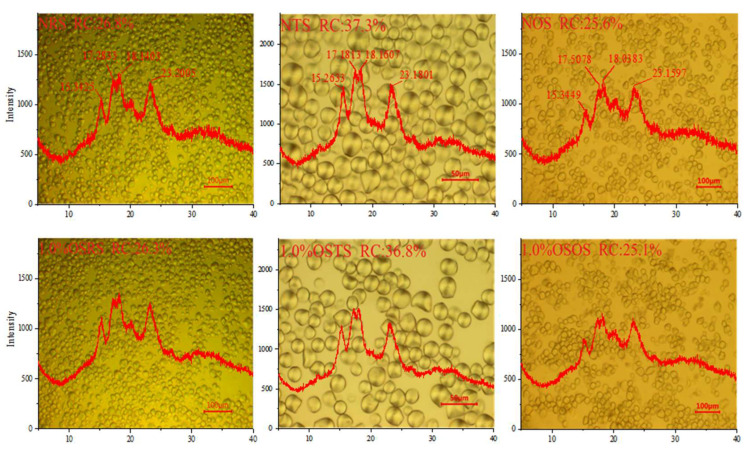

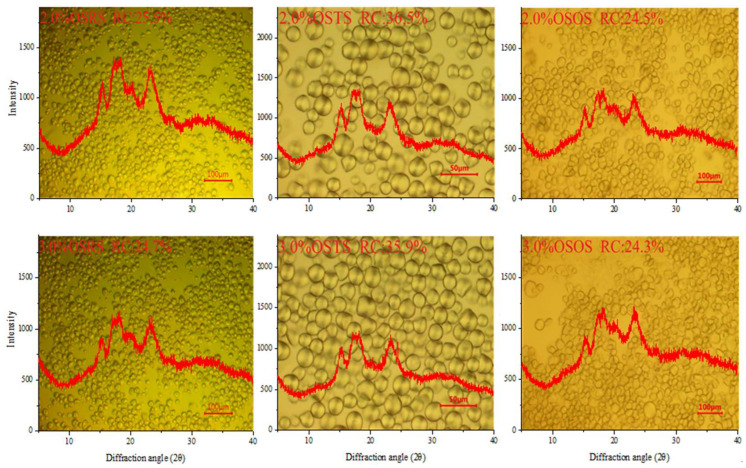
PLM and XRD of NS and OS-starch.

The crystalline structures and amorphous structures of starch differ in their density and refractive index in the starch granules. A polarized cross of the crystalline structure can be clearly observed through the irradiation of polarized light. When the crystalline structures changes, the polarized cross also changes. The polarized crosses of NS (NRS, NTS, NOS) and OS-starch (OSRS, OSTS, OSOS) granules were clearly observed from the PLM of Figure 5; this indicated that both NS and OS-starch had a crystalline structure, and the RC values of NRS, NTS and NOS were 26.8%, 37.3% and 25.6%, respectively. In addition, the XRD diffraction curves of starch will present a specific type of curve, providing sufficient evidence for the orderly crystalline structure of starch granules, that is, the crystalline structure has a sharp peak diffraction characteristic, while the amorphous structure has a diffuse peak diffraction characteristic. The crystalline structure of starch can be classified according to the outline of the XRD diffraction curve, which can be generally divided into four types: A, B, C and V [25,26]. As can be seen in Figure 5, the XRD curves of NRS, NTS and NOS showed strong diffraction peaks near the 2θ = 15°, 17°, 18° and 23° and the continuous peaks at 17°and 18°, which all belong to the characteristic peaks of type A starch [27,28], and the XRD curves of OSRS, OSTS and OSOS also showed characteristic peaks at the corresponding 2θ angular positions, indicating that crystal structure types of the OSRS, OSTS and OSOS still belonged to type A. He et al. [29] suggested that the esterification of rice starch and OSA did not change the crystalline patterns of rice starch. According to Jiang et al. [21], the esterification reaction of taro starch with OSA mainly occurred in the amorphous region of the starch because the OSA modification treatment did not change the crystal structure type of taro starch. However, with the increasing levels of OSA modification and DS values, the RC values of OSRS, OSTS, and OSOS showed a decrease trend, and the intensity and sharpness of diffraction peak were also weakened. Peng et al. [30] and Yu et al. [16] obtained similar results by performing a hydrophobic modification with waxy corn starch and taro starch using OSA, respectively. During the esterification, the OS groups can penetrate into the crystalline region of the starch granules and react with the starch molecules therein and replace the hydroxyl group on the glucose molecule, causing changes in the ordered structure within the starch molecule, thus affecting the RC of the starch granules to a certain extent [31]. These indicated that although the esterification reaction had some effect on the crystalline region of starch, it did not change the crystal structure type of starch, therefore, the esterification reaction mainly occurred in amorphous regions of starch.

#### 3.1.4. Particle Wettability

**Figure 6:**
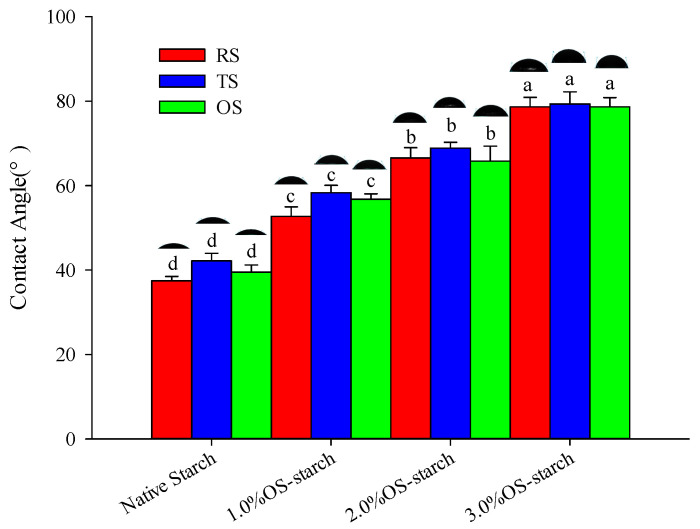
Contact angle of NS and OS-starch. Note: The data means average value (*n* = 3); the different letters (a–d) means differ significantly (*p* < 0.05), the same indicates that the difference is not significant (*p* > 0.05).

Particles’ wettability is usually characterized by the contact angle (θ) of the particles and the aqueous or oil phase; the size of the θ can reflect the amphiphilic properties of the particles as well as the type of emulsion formed. When the θ approaches 0°or 180°, the particles are completely dispersed in the aqueous or oil phase, making it difficult to adsorb at the oil-water interface to form a stable emulsion system [9]. However, when the θ approaches 90°, the particles show a strong amphipathicity and can stably adsorb at the oil-water interface, which, of the emulsions formed, has the highest stability [32]. Generally, the particles tend to form an oil/water (O/W) type of emulsion when the 15° < θ < 90° and tend to form a water/oil(W/O) type of emulsion when the 90° < θ < 165° [33]. The θ (38.45°–82.44°) of NS (NRS, NTS and NOS) and OS-starch (OSRS, OSTS and OSOS) particles (Figure 6) were all below 90°, indicating that these starch particles prefer to form the O/W type of emulsion as particle emulsifier. With the increasing level of OSA modification, the θ of OSRS, OSTS and OSOS also significantly increased and was closer to 90°, which indicated that the tendency of the oil to wet the surface of the starch granules increased, and the hydrophobicity of the starch granules were also enhanced. These changes contributed to the formation of a stable Pickering emulsion by starch as an emulsifier.

### 3.2. Properties of Pickering Emulsions Stabilized by NS and OS-Starch

#### 3.2.1. Emulsions and Emulsion Index (EI)

**Figure 7:**
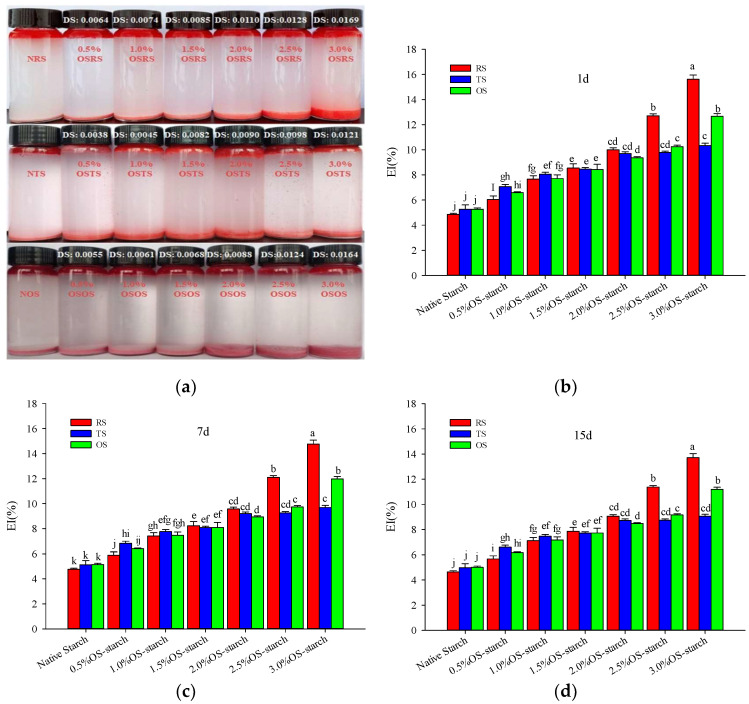
Pickering emulsions prepared from the native starch and OS-starch (**a**), and the EI values of the emulsions standed for 1 d, 7 d and 15 d (**b**–**d**). Note: The data means average value (*n* = 3); the different letters (a–j) means differ significantly (*p* < 0.05), the same indicates that the difference is not significant (*p* > 0.05).

The size of EI values can represent the emulsifying activity of the polysaccharide macromolecular emulsifier, and the greater the emulsion EI value, the better the emulsifying activity of the emulsifier [34,35]. The Pickering emulsions in Figure 7a were prepared from NS and OS-starch with different DS values. The oil in the emulsion was stained with Sudan red, with the aim to more intuitively observe the combined effect of starch and oil. Furthermore, the deposition of these Pickering emulsions droplets at the bottom of the glass bottle was due to the starch/oil ratio (200 mg/mL). The relatively low proportion of the oil in the emulsions caused the relatively dense starch granules to coprecipitate together to the bottom with the oil, during the formation of the emulsion droplets, which was beneficial to observe the amount of starch bound to the oil in the emulsion and the ability of distinguishing between the different types of starch to form the emulsion droplets. Similar findings were obtained for the preparation of Pickering emulsion using quinoa starch (214 mg/oil) by Rayner et al. [22]. It can be seen that the amount of NS bound to the oil in the emulsions was significantly lower than the respective modified OS-starch; the amount of NRS bound oil was the least (red color of the precipitation at the bottom of the glass bottle was the lightest). This may be due to the stronger hydrophilicity of the NRS with the smallest granularity and the largest surface area and having a much greater ability to bind to water in the emulsion than to the oil. Therefore, NRS, NTS and NOS have a low ability to bind as a granular emulsifier to the oil and were not suitable to stabilize the Pickering emulsion. With the increase of starch DS value, the red color of precipitation at the bottom of the glass bottle gradually deepened, and the height of the emulsion layer gradually increased. This shows that the amount of oil bound to the OS-starch in the emulsion significantly increased, and the emulsifying activity of the OS-starch as particle emulsifier were improved. Among them, the Pickering emulsion prepared by 3.0% OSRS had the highest bottom precipitation height and the deepest red color, which indicated that the 3.0% OSRS had the relatively highest emulsifying activity.

Figure 7b–d were the EI values of Pickering emulsions standed for 1 d, 7 d and 15 d, respectively. The EI values were all significantly increased with the increase of DS values. There were no significant differences in the EI between the OSRS, OSTS, and OSOS in the range of 0.5–2.0% OSA treatment levels. However, the EI of OSRS was more significantly increased than OSTS and OSOS in the range of 2.5–3.0% OSA treatment levels. The size order of the EI was 3.0% OSRS > 3.0% OSOS > 3.0% OSTS (*p* < 0.05) at the 3.0% OSA treatment level. These may be related to the particle size and relative crystallinity of starch granules. The tapioca starch has a large particle size and relative crystallinity, while the rice and oat starch have a small grain size and relative crystallinity. When OSA had contact with the same mass of the starch during esterification reaction, the small starch granules had a large contact area with OSA, while the large starch granules had a small contact area with OSA. This was also correlated with the DS and RE of OSRS, OSTS and OSOS. The larger the contact area, the higher the reaction efficiency and thus the higher the DS. On the other hand, because the esterification reaction mainly occurs in the amorphous region of the starch granules, the higher crystallinity may limit the efficiency of the esterification reaction and will also cause the DS and RE of tapioca starch to be lower than that of rice and oat starch. The lower DS value indicates that a smaller number of hydroxyl groups were replaced by OS groups in starch molecules, and the ability to bind the oil in the emulsion was weaker. Thus, the Pickering emulsions prepared with 2.5–3.0% OSRS exhibited relatively higher EI values and stability.

#### 3.2.2. Optical Micrography and Droplet Size Distribution (DSD)

**Figure 8:**
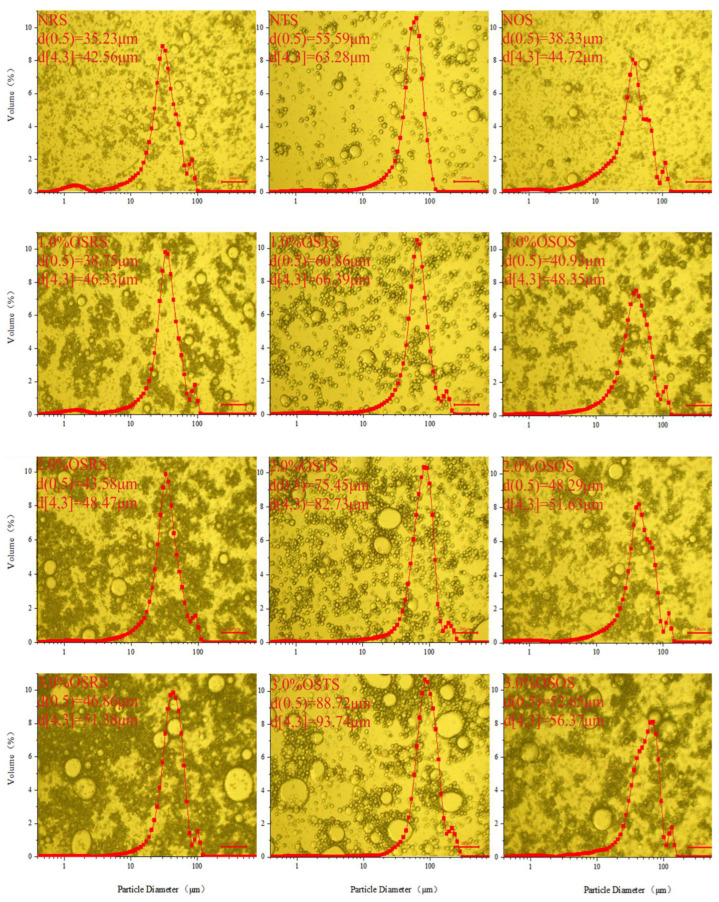
PLM and DSD of Pickering emulsions prepared by NS and OS-starch.

The size and DSD of the emulsion droplets can affect the stability of emulsion. In general, the emulsion droplets with a relatively smaller particle size can be obtained by using a traditional emulsion, such as Tween 20 [22]. The smaller the particle size of the droplet, the more stable the emulsification system was. While the starch granules belong to the polysaccharide macromolecules, which of the way of stabilizing the emulsion was different from that of the traditional emulsifier. The DSD and microstructure of Pickering emulsions prepared by NS and OS-starch as shown in Figure 8. It can be clearly observed that droplet size of emulsions prepared by NRS, NTS and NOS were relatively small, and had a certain amount of free starch distribution in the emulsion. Although some of the starch granules bound together at the droplet interface, the droplets were not wrapped by the starch granules, which were detrimental to limiting droplet aggregation. These indicated that NRS, NTS and NOS have a limited hydrophobicity and a weak ability to stabilize the emulsion.

With the improvement of the OSA modification level, the binding effect of OS-starch to droplets in the emulsion were significantly enhanced. It was observed that the size of the emulsion droplets and the number and thickness of the starch granules on the oil-water interface increased with the improvement of OSA modification levels, and around the droplets in the Pickering emulsion prepared with 3.0% OSRS bound the most starch granules, followed by 3.0% OSOS and 3.0% OSTS, respectively. In addition, an aggregation state was formed between the OS-starch granules, which was also enhanced with the OSA modification levels, and a continuous phase (dark area around the oil droplets) was even formed by starch granules around the Pickering emulsion droplets prepared by 3.0% OSRS. Emulsion droplet wrapped by granular emulsifier and the granular emulsifier aggregation around the droplet to form a certain continuous phase was beneficial to stabilize the Pickering emulsion [36].

The distribution of the emulsion droplets has a clear correlation with the results observed in the micrographs. d(0.5) and d[4,3] of the emulsion droplets increased with increasing of OSA modification levels, and the main peak of DSD also shifted toward a larger droplet size with increasing of OSA modification levels. The emulsions prepared from NRS, NTS, and NOS had weaker peaks, representing free starch granules at 0.72–2.6 μm,1.14–3.51 μm and 0.86–2.21 μm. These weaker peaks gradually disappeared with the improvement of starch OSA modification levels, which indicated that the increased level of OSA treatment causes more starch granules to be adsorbed at the emulsion droplet interface or aggregated between starch granules.

Comparisons made with different NS and OS-starch showed that the smallest d(0.5) and d[4,3] of Pickering emulsion droplets were prepared from NRS and OSRS, followed by NOS and OSOS, and the relatively largest d(0.5) and d[4,3] were prepared from NTS and OSTS. These were related to the particle size of the three types of starch; the small particle size starch having a small interface tension at the oil-water interface and being more easily adsorbed at the oil-water interface after hydrophobic modification, which was the reason for the relatively highest EI value of Pickering emulsion prepared from 3.0% OSRS. Rayner et al. [22] prepared emulsions using quinoa starch (OSA modified) and Tween 20; the former had a larger particle size (d[4,3] = 40.2–50.6 μm) than the latter (d[4,3] = 8.7 μm), and the particle size of Pickering emulsion prepared from quinoa starch also increased with the increasing of OSA modification level. They suggested that higher levels of modification preferred quinoa starch to aggregate with each other in the emulsion and to form a thicker multigranular layer on the droplet surface, caused a shift in the volume frequency distribution to larger diameters, thus improving the stability of the emulsion. Therefore, it can be considered that the ability of OS-starch to stabilize Pickering emulsion was enhanced after a higher level (2.5% and 3.0% OSA) of hydrophobic modification; the emulsification abilities at the same modification level were OSRS > OSTS > OSOS, respectively.

## 4. Conclusions

OSA modified treatment effectively improved the emulsification capacity of NRS, NTS and NOS but did not change the crystal structure type of starch granules. Emulsification ability of OSRS, OSTS and OSOS were affected by the DS value and were related to the size and relative crystallinity of the starch granules. The OS-starch as particle emulsifier stabilized Pickering emulsion depended on starch granules forming a thicker multi-granule layer at the interface of the emulsion droplet, and the starch granules inclined to aggregation each other around the droplet; this trend shifted the particle size distribution of the emulsion droplet to a larger size. Based on the above research results, we plan continue to study the effect of OS-starch based Pickering emulsion on emulsifying and gel properties of myofibrillar protein to provide a reliable theoretical basis for the application of OS-starch as a better food particle emulsifier in meat products.

## Data Availability

The authors confirm that the data supporting the findings of this study are available within the article.
